# BUSYNESS MAY NOT MATTER: ASSOCIATIONS BETWEEN BUSYNESS, ACTIVITY ENGAGEMENT, AND COGNITION

**DOI:** 10.1093/geroni/igae098.2647

**Published:** 2024-12-31

**Authors:** Allison Bielak

**Affiliations:** Colorado State University, Fort Collins, Colorado, United States

## Abstract

Participating in a greater variety and frequency of activities is associated with higher cognitive performance across adulthood. A potentially related metric is perceived busyness (Martin & Park, 2003), defined as scheduled activity patterns. Few studies have investigated this metric, with one finding that those who rated themselves as busier performed higher on a range of cognitive tests (Festini et al., 2016). The present study aimed to answer several questions: 1) Does the link between busyness and cognition replicate?; 2) Does stress interact with busyness in predicting cognition?; 3) How similar is busyness to frequency and variety of activity engagement?; 4) Does busyness predict cognition better than activity variety or frequency? Adults aged 60–92 years (M=70.5, n=206) completed questions related to busyness (in a typical week, how busy is your schedule), stress towards busyness, activity based on past 2-year participation, and cognitive tests. Hierarchical linear regressions controlling for age and education showed busyness was not a significant predictor of processing speed, episodic memory, vocabulary, executive functioning, or reasoning, but did significantly predict working memory (Std B=-.16). Stress about busyness did not interact with busyness for any cognitive domain. Busyness was weakly correlated with activity variety and frequency (rs=.20) and did not significantly predict cognition beyond the activity variables. Although a simple measure of busyness was used, greater busyness was not related to higher cognition. Busyness was only weakly related to the activity indices, and findings suggest that activity variety and frequency might be more valuable measures in evaluating cognition.

